# A prime/boost strategy using DNA/fowlpox recombinants expressing the genetically attenuated E6 protein as a putative vaccine against HPV-16-associated cancers

**DOI:** 10.1186/s12967-015-0437-9

**Published:** 2015-03-05

**Authors:** Massimiliano Bissa, Elena Illiano, Sole Pacchioni, Francesca Paolini, Carlo Zanotto, Carlo De Giuli Morghen, Silvia Massa, Rosella Franconi, Antonia Radaelli, Aldo Venuti

**Affiliations:** Department of Pharmacological and Biomolecular Sciences, Università di Milano, Milan, Italy; Department of Medical Biotechnologies and Translational Medicine, Università di Milano, Milan, Italy; Laboratory of Virology HPV-UNIT, Regina Elena National Cancer Institute, Rome, Italy; Cellular and Molecular Pharmacology Section, CNR Institute of Neurosciences, Università di Milano, Milan, Italy; Technical Unit of Radiation Biology and Human Health, Italian National Agency for New Technologies, Energy and Sustainable Economic Development (ENEA), Casaccia Research Centre, Rome, Italy

**Keywords:** HPV, Recombinant preventive/therapeutic vaccines, Fowlpox virus, Prime/boost immunizations, Mutated E6 oncoprotein

## Abstract

**Background:**

Considering the high number of new cases of cervical cancer each year that are caused by human papilloma viruses (HPVs), the development of an effective vaccine for prevention and therapy of HPV-associated cancers, and in particular against the high-risk HPV-16 genotype, remains a priority. Vaccines expressing the E6 and E7 proteins that are detectable in all HPV-positive pre-cancerous and cancer cells might support the treatment of HPV-related lesions and clear already established tumors.

**Methods:**

In this study, DNA and fowlpox virus recombinants expressing the E6_F47R_ mutant of the HPV-16 E6 oncoprotein were generated, and their correct expression verified by RT-PCR, Western blotting and immunofluorescence. Immunization protocols were tested in a preventive or therapeutic pre-clinical mouse model of HPV-16 tumorigenicity using heterologous (DNA/FP) or homologous (DNA/DNA and FP/FP) prime/boost regimens. The immune responses and therapeutic efficacy were evaluated by ELISA, ELISPOT assays, and challenge with TC-1* cells.

**Results:**

In the preventive protocol, while an anti-E6-specific humoral response was just detectable, a specific CD8^+^ cytotoxic T-cell response was elicited in immunized mice. After the challenge, there was a delay in cancer appearance and a significant reduction of tumor volume in the two groups of E6-immunized mice, thus confirming the pivotal role of the CD8^+^ T-cell response in the control of tumor growth in the absence of E6-specific antibodies. In the therapeutic protocol, *in-vivo* experiments resulted in a higher number of tumor-free mice after the homologous DNA/DNA or heterologous DNA/FP immunization.

**Conclusions:**

These data establish a preliminary indication for the prevention and treatment of HPV-related tumors by the use of DNA and avipox constructs as safe and effective immunogens following a prime/boost strategy. The combined use of recombinants expressing both E6 and E7 proteins might improve the antitumor efficacy, and should represent an important approach to control HPV-associated cancers.

## Background

High-risk human papillomaviruses (HPVs) are the causative agents of cervical tumors, and are responsible for an increasing frequency of anogenital and head and neck tumors [1]. Although different HPV types are considered as cancer-related agents [2], HPV-16 and HPV-18 are the most frequent genotypes, and they are involved in about 62.6% and 15.7% of cervical neoplasias, respectively [3,4].

Preventive vaccines are the first choice for intervention against HPVs, as they can hamper the primary infection by eliciting neutralizing antibodies against the incoming virus, and HPV virus-like-particles (VLPs) have already been produced in different cell-based systems, which are morphologically and immunologically similar to the native virions [5,6]. These VLPs have proven to be effective as prophylactic bivalent (Cervarix®) [7] and quadrivalent (Gardasil®) [8] HPV vaccines. However, no therapeutic activity has been demonstrated against already-established HPV-16 and HPV-18 genital lesions, and the long delay before tumor development limits the assessment of their efficacy in lowering HPV tumor incidence [9]. Moreover, VLPs are type-specific and they generate a limited cross-protection against the genotypes not included in the vaccine. There is therefore a pressing need for novel immune approaches to prevent HPV-related tumors.

The two major HPV oncogenes, E6 and E7, show high levels of protein expression [10,11] that can be maintained for many years. They both cooperate in the immortalization of primary human keratinocytes [12], which are the natural target cells of HPV *in vivo*, and their combined activity is essential for the transformed cell phenotype and the evasion of apoptosis [13,14]. They represent therefore ideal targets for immunotherapy, as they contribute to the progression from initial lesions to malignancy and are constitutively expressed in HPV-associated cancers throughout the replicative cycle of the virus. In particular, while E7 appears to be the main promoter of tumor formation, E6 is required to enhance some of the more malignant aspects of tumorigenesis [15].

Pre-clinical animal models already exist for E6/E7-based vaccines, that can be utilized to design immunogens and eradicate established tumors by challenging mice with syngeneic tumor cells expressing these antigens [16]. These therapeutic vaccines mainly aim at generating a T-cell-specific immunity, rather than a humoral response, by stimulating antigen presentation to CD8^+^ cytotoxic T lymphocytes (CTLs), and CD4^+^ T helper T cells. When expressed as an L2/E6/E7 fusion protein by genetic DNA vaccines, they already proved to be able to control tumor growth [17,18] through the induction of CTLs targeted to cancer cells [19-21]. DNA-based vaccines have emerged as an attractive approach, because of their safety and stability, and the possibility of their repeated administration. They are also an efficient means to target dendritic cells, and thus to prime antigen-specific CD4^+^ and CD8^+^ T-cell immune responses *in vivo* [22].

Due to their high immunogenicity, live recombinant viral vectors have also been used as HPV vaccines, as they facilitate the spread of antigens. These vaccines have been explored in pre-clinical models [23,24], where they showed protective and therapeutic antitumor effects against E7-expressing tumors in vaccinated mice. They were safe, well tolerated and could stimulate antigen-specific antibody and CTL responses [25-28]. The attenuated modified vaccinia Ankara (MVA) co-expressing E6/E7 and IL-2 as an adjuvant has also been shown to be effective in Phase II clinical trials [29,30], but did not enter into Phase III.

Viral recombinants have also been assessed using heterologous prime/boost regimens to enhance their immunogenicity and to limit the induction of neutralizing antibodies against the vector. Live virus-based recombinant vaccines, either as VV, MVA [31,32] or adenoviruses [33], can be successfully primed by E6/E7 DNA-based genetic vaccines. Conversely, the use of the E6/E7 fusion proteins to either prime or boost VV-based HPV vaccines did not show any correlation between immunological and clinical responses [28,34]. However, as MVA replication in mammals is only partially abortive [35,36], the search for alternative safe vectors is still ongoing.

Canarypox and fowlpox (FP) avian poxviruses have been developed as novel recombinant vectors against human infectious diseases, and as vaccines against HPV in preclinical [37], but not clinical, studies. As their replication is restricted to avian species [38], they represent safe immunogens that are permissive for entry and transgene expression in most mammalian cells [39,40]. Avipoxviruses are also immunologically non cross-reactive with VV, and can thus escape pre-existing immunity in smallpox-experienced humans.

An *in-vivo* single-point E6 mutant of HPV-16, E6_F47R_, has also been identified as defective for polyubiquitination and degradation of p53, which competes with the endogeneous E6 [41]. By hampering the p53 degradation both *in vitro* and *in vivo*, E6_F47R_ changes the E6 oncoprotein into a suppressor of proliferation of HPV-positive HeLa cells [42].

The aim of the present study was to generate preventive/therapeutic DNA-based and FP-based recombinant vaccines that express the modified HPV-16 E6_F47R_ gene, and to determine their immunogenicity and efficacy by different prime/boost immunization protocols. Their effects were evaluated in the C57BL/6 mouse model of HPV-related tumorigenesis by immunizing groups of mice before and after the challenge with TC-1* syngeneic cancer cells. The animals were monitored for E6-specific humoral and cellular immune responses, and for the control of tumor growth and survival to establish the immune correlates of protection and tumor reduction in the immunized animals.

## Methods

### Cells

Specific-pathogen-free primary chick embryo fibroblasts (CEFs) were grown in Dulbecco’s modified Eagle’s medium supplemented with 5% heat-inactivated calf serum (Gibco Life Technologies, Grand Island, NY, USA), 5% tryptose phosphate broth (Difco Laboratories, Detroit, MI, USA), 100 U/ml penicillin and 100 mg/ml streptomycin. CaSki cells, which contain multiple copies of integrated HPV-16 DNA, green monkey kidney (Vero) cells, normal human lung fibroblasts (MRC-5), and TC-1 star (TC-1*) mouse cells (which constitutively express the HPV-16 E6 and E7 oncoproteins, but are more aggressive than TC-1 and have been shown to induce tumors in 100% of the injected mice [43]), were grown in Dulbecco’s modified Eagle’s medium supplemented with 10% calf serum and 100 U/ml penicillin and 100 mg/ml streptomycin.

### Construction of the pDNAE6_F47R_ expression plasmid

The pDNAE6_F47R_ expression plasmid was used for the mice immunizations, and it contains the mutated E6_F47R_ sequence of the HPV-16 E6 gene [41,42]. The pX5-E6-F47R/6C6S plasmid that contains the mutated E6_F47R_ gene was a kind gift from G. Travé (CNRS, University of Strasbourg, Illkirch, France). This mutant was obtained by replacing one phenylalanine (F) with one arginine residue (R), and six cysteine (C) with six serine (S) residues. The first mutation prevents p53 degradation, whereas the C/S substitutions were introduced to minimize oxidation and to stabilize the protein. Briefly, after excision from the pX5-E6-F47R/6C6S plasmid using the EcoRI and NotI enzymes, the E6-F47R/6C6S gene was inserted into the pcDNA3 plasmid (Life Technologies Corp., Carlsbad, CA, USA). This plasmid was propagated in *Escherichia coli* XL1-Blue and extracted by alkaline lysis, followed by plasmid purification with endotoxin removal (Qiagen, EndoFree Plasmid Giga Kit, Hilden, Germany). After dissolving this plasmid in Ca^2+^-free and Mg^2+^-free phosphate-buffered saline (PBS^−^) to a final concentration of 1 mg/ml, this was used for immunization of the mice.

### Construction of the FPE6_F47R_ recombinant virus

The recombinant FP virus expressing the E6_F47R_ protein (FPE6_F47R_) was obtained by *in-vitro* homologous recombination [44]. Briefly, the genetically mutated E6_F47R_ gene of HPV-16 was amplified by PCR from the pcDNA3E6_F47R_ plasmid and inserted downstream of the VVH6 vaccinia virus early/late promoter into the pFP_MCS_ vector, which contained the 3-β-hydroxysteroid dehydrogenase 5-delta 4 isomerase gene and was interrupted by a multiple cloning site [45]. The DNA sequence that encodes the E6_F47R_ region was amplified using the forward V364 (5′ CCG CGC CCG GGA AGC TTA TGC ACC AAA AGA GAA CT 3′) and the reverse V99 (5′ CGA AGC TTT TAC AGC TGG GTT TCT CTA CG 3′) primers. The amplification was carried out as described previously [46]. The plasmid DNA was purified and the E6_F47R_ was sequenced (Bio-Fab Research, Rome, Italy) to exclude any possible mutations arising from the PCR amplification and designated as pFPE6_F47R_ (8,700 bp). The FPE6_F47R_ recombinant was obtained by *in-vitro* recombination on primary CEFs, as described previously [47], using FP wild-type (FPwt) and pFPE6_47R_ [48]. The recombinant clones were identified by autoradiography after hybridization with the [^32^P]-labeled E6_F47R_ probe, picked, and subjected to multiple cycles of plaque purification. One clone was selected for its correct and high expression of the E6_F47R_ gene, as determined by RT-PCR, Western blotting and immunofluorescence using anti-E6-specific antibodies. The FPE6_F47R_ recombinant virus was amplified in CEFs, purified on a discontinuous sucrose gradient, titered, and used for mice immunization.

### RT-PCR

Expression of the E6_F47R_ gene in CEFs and Vero and MRC-5 cells was investigated first using RT-PCR. The cells were infected with one plaque-forming unit (PFU)/cell of the FPE6_F47R_ virus. Total RNA extraction was carried out 24h post-infection, using Trizol LS (Gibco, Life Technologies), and the mRNAs from all of the samples were prepared as described previously [49]. Briefly, 50 ng RNA from each sample was used in a final volume of 10 μl, in the presence of 1 μM of each primer, 200 mM of each dNTP, 0.1 U/μl *Thermus flavus* DNA polymerase, 0.1 U/μl avian myeloblastosis virus reverse transcriptase, and 2.5 mM MgSO_4_. The V348 (5′ CTG CAA TGT TTC AGG ACC 3′) and V99 primers were used. The reverse transcriptase reaction was performed at 48°C for 45 min, followed by 2 min at 94°C. PCR amplification was carried out for 40 cycles at 94°C for 30 s, 55°C for 30 s, and 68°C for 30 s, followed by a final incubation at 68°C for 7 min. FPwt-infected cells were used as negative controls, and the pFPE6_F47R_ plasmid was used as the positive control.

### Western blotting

To determine whether the E6_F47R_ protein was expressed correctly, CEFs and Vero and MRC-5 cells were infected with 10 PFU/cell FPE6_F47R_ and examined by Western blotting, as described previously [46]. The blotted nitrocellulose membranes were incubated overnight at 4°C with 1:100 dilution of the primary rabbit AbE6/Mu polyclonal antibody (M. Mueller, German Cancer Research Center, Heidelberg, Germany). After a 1-h incubation with a goat anti-rabbit horseradish peroxidase (HRP) antibody (1:2000 dilution; DakoCytomation, Carpinteria, CA, USA) and 2-h washes, the proteins were revealed using the ECL system (GE Healthcare, Buckinghamshire, UK). Cells infected with FPwt were used as the negative control, and the E6 protein as the positive control.

### Immunofluorescence

To detect the expression of the E6_F47R_ protein, immunofluorescence was also carried out on CEFs and Vero and MRC-5 cells infected with 5 PFU/cell, as described previously [48]. The expression of the E6_F47R_ gene was also verified after transfection with the pcDNA3E6_F47R_ plasmid by the PolyJet™ transfection reagent (SignaGen Lab., Rockville, MD, USA). Briefly, cells were overlaid with 1 μg reagent in 1.1 ml medium per 4-cm-diameter Petri dish without serum and antibiotics for 16–24 h, and replaced with the complete medium for an additional 24 h. The samples were then treated for 1 h with the 1:100-diluted mouse AbE6/Gi or with the 1:300-diluted rabbit Rpool polyclonal antibodies, obtained in our laboratory [50] followed by the 1:100-diluted FITC-labeled goat anti-mouse or sheep anti-rabbit sera (DakoCytomation) for 1 h. FPwt and pcDNA3 empty plasmid were used as the negative controls to infect or transfect the different cell lines. The samples were viewed under a Zeiss Axioskop fluorescence microscope.

### Production of wild-type E6 protein in its native form in bacteria

The pQE30 expression plasmid (Qiagen) was engineered to contain the E6 gene of HPV-16, which is referred to as pQE30-E6/His, inserted in *E. coli* (strain M15, Qiagen), and the protein was prepared under non-denaturing conditions. Briefly, after 16h of growth, the culture was 100-fold diluted in fresh Luria-Bertani broth with 25 μg/ml kanamycin and 100 μg/ml ampicillin, and incubated at 37°C until an OD_600_ of 0.6-0.7 was reached. Protein expression was induced with 1 mM isopropyl-β-D-thiogalactopyranoside. The cells were maintained at 28°C for 16 h, and then harvested by centrifugation at 4,000× *g* for 20 min at 4°C. The pellet was resuspended in lysis buffer (50 mM NaH_2_PO_4_, 200 mM NaCl, 20 mM imidazole, 100 μM dithiothreitol [DTT], pH 7.5) containing an EDTA-free anti-protease cocktail (Roche, Basel, Switzerland) at the concentration recommended by the manufacturer. After adding 1 mg/ml lysozyme and 1% Triton X-100, the cells were incubated for 1 h at 4°C and then sonicated on ice at a 10-Hz output (3 times for 1 min). Clarification was performed by centrifugation at 15,000*× g* for 45 min at 4°C, and the supernatant was incubated for 16 h with 1 ml Ni-nitrilotriacetic acid agarose resin (Qiagen) that had been pre-equilibrated in lysis buffer. After washing several times with 50 mM NaH_2_PO_4_, 200 mM NaCl, 70 mM imidazole, 100 μM DTT, pH 7.5, to a final OD_280_ of 0.01, the protein was eluted in different fractions using 50 mM NaH_2_PO_4_, 200 mM NaCl, 300 mM imidazole, 100 μM DTT, pH 7.5, and run on 12% SDS-PAGE. The fractions enriched in the recombinant proteins were pooled, quantified, and stored at 4°C until use. The purified E6 protein (pE6) was dialyzed in PBS^−^ containing betaine (0.02%) and DTT (100 μM), and used for the enzyme-linked immunosorbent assays (ELISA).

### Immunization protocols and challenge with TC-1* tumor cells

**Figure 1:**
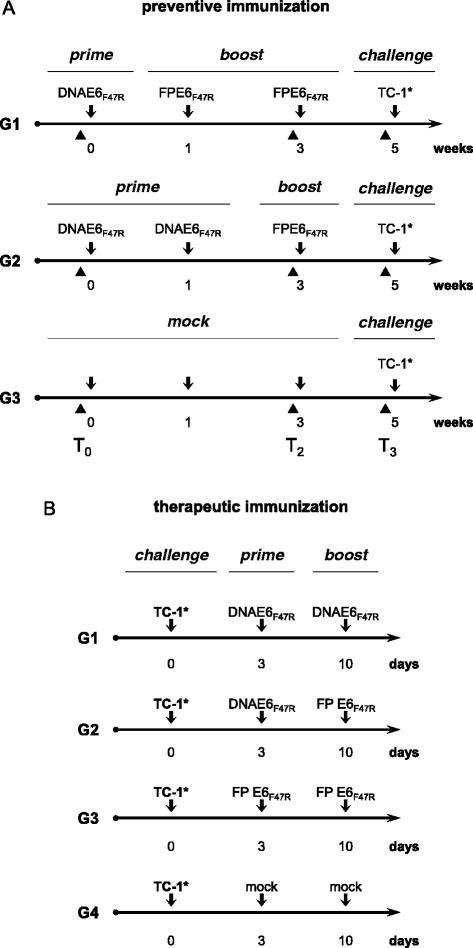
**Immunization protocols.** For the preventive response **(A)**, six mice per group were used to evaluate the immune responses and the protection of the vaccinated mice. Half of the mice for each group were challenged with syngeneic tumor TC-1* cells, while the remaining mice were used for spleen removal and ELISPOT assays for the cell-mediated immune responses. For the therapeutic immunization **(B)**, five mice were challenged with TC-1* cells, primed once three days after challenge, and boosted one week after the priming. Black triangles, times of mice bleeding; arrows, vaccine administration and challenge.

In the preventive protocol (Figure 1A), three groups of six 6-week-old female C57BL/6 mice (Charles River Laboratories, Como, Italy) were vaccinated by multiple injections. The mice were primed with the recombinant pDNAE6_F47R_ plasmid once (protocol G1; 100 μg/mouse, intramuscular, i.m.) or twice (protocol G2; 100 μg/mouse, i.m.). Two weeks after the DNA inoculation, they were boosted twice (protocol G1) or once (protocol G2) with FPE6_F47R_ (10^7^ PFU/mouse, subcutaneous, s.c.). All of the mice remained in good health, and, after the last immunization, half of the mice of each group were sacrificed to perform the enzyme-linked immunospot (ELISPOT) assays. All of the other mice were challenged by a s.c. injection of 5 × 10^3^ E6/E7-expressing TC-1* syngeneic tumor cells in 200 μl saline solution [43]. The control mice (Figure 1, protocol G3) were repeatedly mock infected and tumor growth was monitored twice a week until tumor appearance, and every day thereafter by visual inspection and palpation.

In the therapeutic protocol (Figure 1B), four groups of five mice were challenged by s.c. injection with TC-1* cells (5 × 10^3^ in 200 μl saline solution). Three and ten days after challenge, the mice were immunized twice with DNAE6_F47R_ (protocol G1; 100 μg/mouse, i.m.) or with DNAE6_F47R_ followed by FPE6_F47R_ (protocol G2; 10^7^ PFU/mouse, s.c.) or twice with FPE6_F47R_ (protocol G3; 10^7^ PFU/mouse, s.c.). The mice for protocol G4 were mock vaccinated and used as controls. Tumor growth was monitored by visual inspection and palpation twice a week. Animals were scored as tumor-bearing when tumors reached a size of approximately 1 to 2 mm in diameter. Animals were euthanized for ethical reasons when tumors reached a volume of about 4-cm^3^, as calculated by the formula “length × width^2^ × 0.5”. All of the mice were housed and handled under specific-pathogen-free conditions at the Animal House of the Regina Elena Cancer Institute (Rome, Italy) upon the approval of local Ethical Committee, in accordance with the European Guidelines no. 86/609/CEE and 116/92 for the protection of laboratory experimental animals and laboratory animal care (Ministry of Health, Department for Veterinary Public Health, Nutrition and Food Security, Protocol 17/2006).

### ELISA

The ELISA was essentially performed as described previously [51], using either the purified native pE6 protein or the CaSki cells as the plate-bound antigen. Briefly, 96-well maxisorp microtiter plates (Nunc, Naperville, IL, USA) were coated with pE6 (300 ng/well) in PBS^−^ or with CaSki lysates (10^5^ cells/well) in 0.05 M carbonate–bicarbonate buffer, pH 9.6, and incubated overnight at 4°C. CaSki cells were disrupted by freeze-thawing three times and MRC-5 cells were used as a negative control. The sera were then added at a 1:50 dilution, and the binding was revealed by a 1:2000 dilution of goat anti-mouse HRP-conjugated sera (Dako) and tetramethylbenzidine substrate (Sigma-Aldrich Italia, Milan, Italy). The absorbance for each well was measured at 450 nm with a 550 microplate reader (Bio-Rad Laboratories, Hercules, CA, USA).

### ELISPOT assay for interferon-γ-secreting cells

HPV-16 E6-specific T-cell precursors were detected using ELISPOT assays for IFN-γ-secreting cells (BD™ ELISPOT, BD Biosciences Pharmingen, San Diego, CA, USA) [52]. Briefly, the mice were euthanized by cervical dislocation one week after the last immunization. A single-cell suspension of splenocytes harvested from the mice from each group was added to microtiter wells (10^6^ cells/well) that had been pre-coated overnight at 4°C with an anti-mouse-IFN-γ antibody (5 μg/ml; BD Biosciences Pharmingen) along with IL-2 (50 units/ml; Sigma). Stimulation was performed in triplicates at 37°C for 24 h to 72 h, either with three different concentrations of pE6 (0.1, 1.0, 10 μg/ml) or with two E6-specific epitopes (aa 50–57, aa 18–26) [53,54], to detect the T-cell precursors. A mixture of phorbol myristate acetate and ionomycin was used to detect responsive cells. An unrelated protein (scFv), a single chain recombinant antibody produced in our laboratory with a procedure similar to the one used for the E6 protein, was used as a negative control. The plates were incubated with a biotinylated anti-mouse IFN-γ antibody (2 μg/ml; BD Biosciences Pharmingen) for 4 h at room temperature. Streptavidin-HRP was then used for 1 h at room temperature, and the cell spots were stained by addition of 3-amino-9-ethylcarbazole substrate for 1 to 5 min. The spots were counted under a dissecting microscope.

### Statistical analyses

Statistical analyses were performed using one-way ANOVA parametric tests and Bonferroni analysis of variance, using the GraphPad Prism 5 software. Statistical significance was set as p <0.05 (*), p <0.01 (**), p <0.001 (***).

## Results

### The FPE6_F47R_ and the pDNAE6_F47R_ recombinants correctly express the E6_F47R_ transgene

**Figure 2:**
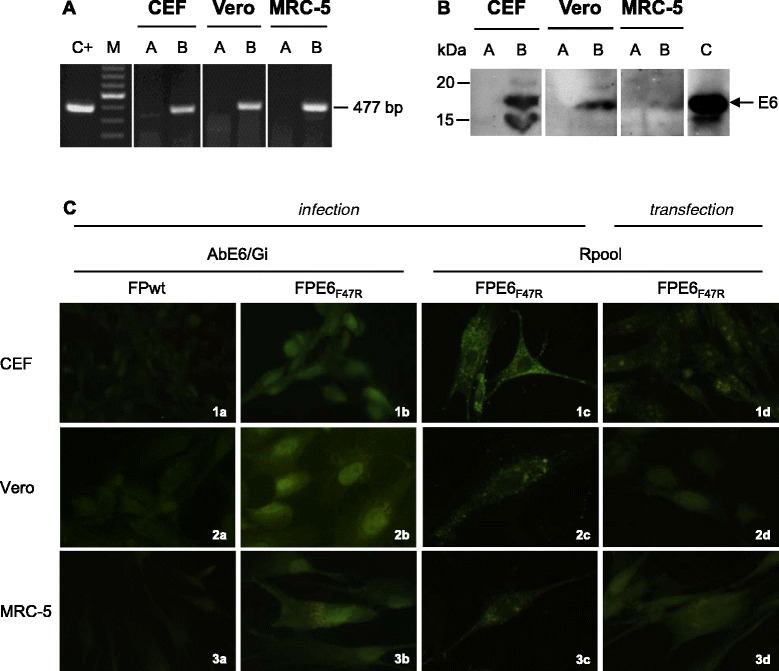
**Expression of the E6**_**F47R**_ **in replication-permissive avian and replication-restrictive mammalian cells.** RT-PCR was used to amplify the 477-bp E6-specific transcripts in all of the cell types (**A**, lanes B). FPwt-infected cells were used as negative controls (**A**, lanes A) and the plasmid pFPE6_F47R_ as a positive control (C^+^). M; 100-bp ladder. Western blotting was used to reveal the presence of a 19-kDa protein in cells infected by FPE6_F47R_ (**B**, lanes B) using the rabbit AbE6/Mu polyclonal antibody. FPwt-infected cells (**B**, lanes A) and the E6 protein (**B**, lane C) were used as a negative or positive controls, respectively. By immunofluorescence **(C)**, specific staining was detectable in all cell lines after infection with FPE6_F47R_ (**C**; 1b, 2b, 3b and 1c, 2c, 3c) or transfection with pDNAE6_F47R_ (**C**; 1d, 2d, 3d) recombinants, using either the mouse AbE6/Gi or the rabbit Rpool polyclonal antibodies. FPwt-infected cells were negative (**C**; 1a, 2a, 3a), as well as the pcDNA3-transfected cells, as expected (data not shown).

RT-PCR of RNAs from FPE6_F47R_-infected CEFs and Vero and MRC-5 cells showed amplification of a 477-bp band (Figure 2A, lanes B) that corresponds to the E6 transcript. The pFPE6_F47R_ plasmid was used as a positive control (Figure 2A, C^+^). FPwt-infected cells were always negative (Figure 2A, lanes A), as well as samples processed in the absence of reverse transcriptase (data not shown).

Western blotting of FPE6_F47R_-infected CEFs and Vero and MRC-5 cell lysates showed a 19-kDa oncoprotein band (Figure 2B, lanes B). No bands were seen when these cells were infected with FPwt (Figure 2B, lanes A). The nonmutated E6 protein produced by an engineered bacterial vector was used as a molecular weight control marker (Figure 2B, lane C). The E6_F47R_ protein was expressed at higher levels by CEFs (5.8-fold) and Vero cells (4.4-fold) than by MRC-5 cells, as determined by densitometric analysis.

**Figure 3:**
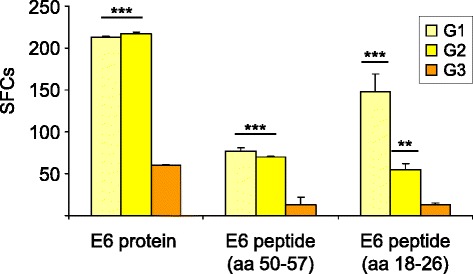
**Functional virus-specific T-cell responses to E6.** IFN-γ production was measured in an ELISPOT assay after preventive immunization to detect CD8^+^ T-cells after specific antigenic stimulation with the E6 protein and peptides. The numbers of IFN-γ-secreting spot-forming cells (SFCs) were determined after subtracting the values obtained using the scFv unrelated protein. Data are presented as fold-increased responses to the E6-specific protein and peptides. G2 and G3 are the protocols for the DNA/FP and FP/FP prime/boost vaccinations, respectively. Statistical significances using the ANOVA parametric test are shown: **p <0.01; ***p <0.001.

After FPE6_F47R_ infection (Figure 2C; 1b, 2b, 3b and 1c, 2c, 3c) or pDNAE6_F47R_ transfection (Figure 2C; 1d, 2d, 3d), immunofluorescence was detectable in CEFs and Vero and MRC-5 cells using either the mouse AbE6/Gi or the rabbit Rpool polyclonal antibodies, with granular nuclear and perinuclear/cytoplasmic localization. FPwt-infected cells were negative, as expected (Figure 2C; 1a, 2a, 3a).

### The E6-specific humoral response is very low in vaccinated mice

The immunized mice were tested for E6-specific humoral immunity. The antibody response against E6 was measured using ELISA, with plates coated with either the native purified recombinant pE6 protein or the CaSki cell lysates. The mice sera were analyzed before the first immunization (T0), after the first boost (T2), and before the challenge (T3), but the antibody responses were generally low, both when using the E6 protein or CaSki cell lysates (data not shown).

### E6-immunized mice show a CD8^+^-specific cellular response

IFN-γ-secreting spot-forming cells (SFCs) were counted using splenocytes obtained after the third immunization from the mice that were not challenged. The mean of the SFCs was assessed after subtracting the number of IFN-γ-positive cells stimulated with the unrelated scFv protein, that can be considered the splenocyte stimulation resulting from the LPS content of the protein. An increase in IFN-γ-producing cells was seen for both of the G1 and G2 immunization protocols (see Figure 1), above the level observed in the mock-vaccinated mice of protocol G3 (Figure 3). In particular, when the splenocytes were stimulated with the E6 protein, protocols G1 and G2 showed an increased response *vs* G3 (p <0.001). When the E6-specific aa 50–57 peptide was used, this increase was even higher for G1 and for G2 *vs* G3 (p <0.001). The same results were obtained when the aa 18–26 peptide was used, with a major increase for G1 (p <0.001 *vs* G3) compared to G2 (p <0.01 *vs* G3).

### Preventive immunization delays tumor appearance and reduces tumor growth

The immunized mice were challenged with syngeneic TC-1* cells two weeks after the last boost, and then regularly examined for tumor growth. Tumor growth first appeared 10 days post-challenge (p.c.) in all of the mice under protocol G3 (Table 1), whereas, in the mice under the G1 and G2 immunization protocols, tumor growth was delayed to 16 days p.c. At 20 days p.c., all of the mice were still alive, although the data on their further survival were not collected thereafter, as they were euthanized for ethical reasons. A clear difference in the tumor volume was found for the cancer-bearing mice that followed protocols G1 and G2 *vs* protocol G3. In particular, the tumor size was significantly lower in the mice that followed protocol G2 *vs* protocol G3 at day 23 p.c. (p <0.05).

**Table 1:** **Inhibition of tumor growth after preventive immunization**

	**Tumor appearance (days p.c.)**	**Mean tumor volume 23 days p.c. (cm** ^**3**^ **± SD)**
**G1**	16	2.52 ± 0.7
**G2**	16	1.96 ± 0.4*
**G3**	10	3.36 ± 0.2

p.c., post challenge.

*p <0.05 G2 *vs* G3, by the one-way ANOVA parametric test.

### Therapeutic immunization with DNA/FP recombinants delays tumor growth

**Figure 4:**
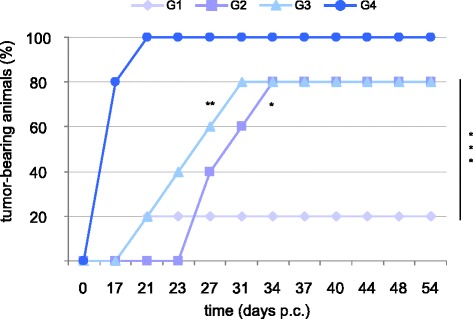
**Tumor growth after therapeutic immunization.** Mice were challenged with TC-1* cells and immunized twice at day 3 and 10 p.c. Immunization was performed with DNA/DNA (G1), DNA/FP (G2), and FP/FP (G3) recombinants expressing the E6_F47R_. The mice of group 4 were mock vaccinated. Tumor growth was monitored by visual inspection and palpation. Animals were euthanized when the tumors reached a 4-cm^3^ volume.

After injecting the TC-1* cells into naïve mice, prime/boost immunizations were performed on days 3 and 10, respectively (Figure 1B). The number of tumor-bearing animals was significantly lower in immunized mice compared to the control (Figure 4, G1, G2, G3 *vs* G4, p <0.001). Tumor appearance was delayed to day 21 p.c. in 20% of mice of G1 and G3, and to day 27 p.c. in 40% of the animals of G2. Tumor development was seen in 80% of the mice at day 17 p.c. in G4, and at day 34 and 31 p.c. in G2 and G3. At day 54, only 20% of the animals of G1 developed tumors, whereas cancer-bearing mice remained 80% in G2 and G3. The numbers of healthy mice remained higher in G1, when compared to both G2 and G3. Statistical analyses also indicate a significant difference between G2 *vs* G1 (p <0.05) and G3 *vs* G1 (p <0.01).

## Discussion

Although most HPV vaccines have to date focused on the E7 oncogene, E6 also represents a target for potential vaccine development to control HPV-associated lesions [14]. E6 is co-expressed with E7 in the majority of HPV-associated tumors, and is responsible for the full malignant transformation through its association with cellular target proteins [1]. However, E6 is poorly immunogenic in the C57BL/6 mouse model and the use of a mutated version of the gene is essential to minimize the safety concerns for DNA and FP vaccines translation to the clinic.

In the present study, we have described the construction of new genetic and FP recombinants that express the mutated non-oncogenic HPV-16 E6_F47R_ protein to be used in combination as a preventive/therapeutic antitumor vaccine. When expressed in HPV-positive cervical cancer cells, E6_F47R_ acts as a dominant-negative mutant by counteracting the p53 degradation activity of the endogenous E6, and thus restores high p53 protein levels [41,42].

After animal immunization, both the humoral and cell-mediated immune responses were evaluated, as well as their correlation with tumor-size reduction or delay in tumor development to prove the therapeutic efficacy. Our results demonstrated that: (i) the pDNAE6_F47R_ as well as FPE6_F47R_ recombinants can correctly express the E6_F47R_ protein in different cell lines; (ii) the CD8^+^ T cell response was higher in the mice immunized with the E6 recombinants using the G1 and G2 protocols; (iii) in the preventive immunization, after challenging with TC-1* cells, tumor appearance was delayed, and the tumor volume was lower at day 23 p.c. in the mice under the G1 and G2 protocols, compared to the G3 control group; (iv) in the therapeutic immunization, the numbers of healthy animals remained higher after the DNA/DNA regimen, but tumor appearance was delayed in all of the protocols and was more evident by the prime/boost DNA/FP schedule.

The amounts of the transcripts expressed by FPE6_F47R_ in all of the cell lines were similar, whereas a different protein expression level was seen by Western blotting. This cannot be ascribed to a lower efficiency of the E6_F47R_ gene expression by the FP vector, as protein expression was evident in all of the infected cells using immunofluorescence. In CEF, as well as in the positive control, an extra-band was seen, also described in other studies [11,55]. Although this might be due to an already observed splicing process [56], this cannot be the case of FP viruses that replicate in the cytoplasm. The presence of an additional ATG, downstream the initial starting codon [11], can in fact result in the synthesis of a lower-MW E6 (151 aa, around 18 kDa) in some cell lines such as in CaSki cells, besides the full length E6 protein (158 aa, around 19 kDa). No significant differences were observed by immunofluorescence after infection or transfection with FPE6_F47R_ or pDNAE6_F47R_.

The numbers of IFN-γ-secreting cells was significantly higher after immunization with the E6 recombinants, which are essential in clearing TC-1* tumor cells. Surprisingly, although the aa 18–26 subdominant epitope may contribute only slightly to the antitumor effect [54], a higher response by the IFN-γ-producing splenocytes was detected to this peptide in the G1 *vs* G2 immunization protocol. HPV-16 E6 epitope mapping [10] revealed that the aa 50–57 region represents the minimal core sequence essential for E6-specific CTL activity, and that the aa 48–57 peptide is the optimal immunodominant sequence. The E6 aa 50–57 deletion prevented protection of TC-1-challenged mice from a DNA vaccine [53,57], and it is to be noted that the E6 aa 48–57 peptide contains CTL epitopes that are presented by E6-expressing TC-1 cells. Although it has still not been determined which HLA-A24-restricted epitope is the most immunogenic one and the most suitable for immunotherapy, a novel potent HPV-16 E6 aa 66–74 peptide has recently been reported, that can be used *in vitro* as an efficient means of CTL induction [57] to discriminate between the different immunization regimens.

After the preventive immunization and the TC-1* challenge, mice remained tumor-free up to 10 days, and tumor appearance was delayed up to 16 days in the animals of groups 1 and 2. Also, when the tumor developed, its volume was significantly smaller compared to the controls, thus indicating the role of the CTL response.

After the therapeutic immunization, tumor growth was also delayed in mice of groups 1, 2, and 3, with 80% of still-healthy animals at 21 days p.c., when 100% of the control animals (group 4) developed tumors. In particular, the numbers of healthy animals remained higher after the DNA/DNA protocol, which should therefore be considered the most effective regimen. However, although genetic vaccination have emerged as an attractive strategy for immunotherapy, in studies performed in non-human and human primates, DNA vaccines suffered from low immunogenicity and efficacy.

Previous studies have also demonstrated that higher protection can be obtained when animals are primed with recombinant DNA followed by recombinant FP boost than when using FP recombinants alone [37,52]. In the present study, we tested both DNA/FP and FP/FP prime/boost immunization regimens. Although the repeated use of DNA for priming (G2) or FP for boost (G1) gave similar results in the preventive immunization, a difference was noted between group 2 and group 3 in the therapeutic immunization when using DNA/FP rather than FP/FP. This suggests that DNA prime followed by the FP boost can be a better strategy to induce CTLs, kill tumor cells, and be effective in eliciting anti-tumor immune responses in humans.

Many HPV vaccine strategies have focused on eliciting HPV E6- and E7-specific T-cell responses, and several vaccination trials have been performed on patients with cervical cancer, genital warts and papillomas [58,59] also to improve antigen processing and presentation [60,61]. In this context, a partial tumor regression was obtained in the rabbit model by VSV-based vaccines encoding the E6 or UbE6 [62,63] and a potent antitumor effect was induced by administering mice with DNA vaccines encoding HPV-16 E6, E7 and L2 proteins fused to calreticulin [61], some of which are also in clinical trial [64]. An E7 DNA vaccine was shown to be ineffective in the TC-1* mouse model, whereas the same gene linked to immunostimulating functions such as the potato virus X coat protein sequence [65] or fused to Zera® peptide [66] showed a strong antitumor activity or tumor regression.

However, vaccines expressing the E6 gene alone have not previously been tested for therapeutic efficacy in prime/boost protocols.

Due to their natural restricted replication to avian species [38], their correct expression of transgenes in mammalian cells, and their ability to elicit a complete immune response in vaccinated hosts [67], FP recombinants represent alternative and safer immunogens than VV, which can cause lytic infection, ulceration, and scab formation. Our observations that a DNA/FP vaccination schedule with E6-based non-adjuvanted vaccine can significantly enhance the E6-specific CD8^+^ T-cell response and partially control the growth of E6-expressing tumor cells strengthen the effectiveness of this prime/boost strategy and the importance of CTL response for tumor inhibition.

## Conclusions

This study aimed at verifying the immunogenicity induced by a preventive vaccine expressing the HPV-16 E6_F47R_ protein and the therapeutic efficacy of different immunization protocols after challenging mice with TC-1* cells. By the preventive immunization protocol, our data show a delay in tumor appearance and a reduction in tumor volume in the two groups of immunized animals and establish the pivotal role of CTLs for tumor inhibition. By the therapeutic immunization, after challenging with TC-1* tumor cells, the number of healthy animals remained higher after the DNA/DNA protocol, but an evident delay of tumor growth was observed using the DNA/FP protocol. Although a better efficacy was expected, it is our hope that the combined use of recombinants expressing both E6 and E7 proteins using the DNA prime/FP boost strategy might improve the antitumor effects. This should represent an important approach to control HPV-associated cancers and we cannot exclude that these vaccines might also be used for therapy in already-infected but still-healthy subjects.

## Abbreviations

CEFs: Chick embryo fibroblasts
CTL: Cytotoxic T cells
ELISA: Enzyme-linked immunosorbent assays
ELISPOT: Enzyme-linked immunospot
FP: Fowlpox
FPwt: FP wild-type
HPVs: Human papilloma viruses
HRP: Horseradish peroxidase
MVA: Modified vaccinia Ankara
p.c.: Post-challenge
PBS^-^: Ca^2+^ −free and Mg^2+^ −free phosphate-buffered saline
PFU: Plaque-forming unit
VLPs: Virus-like-particles
VV: Vaccinia virus

## References

[1] Zur Hausen H (2009). Papillomaviruses in the causation of human cancers: a brief historical account. Virol.

[2] Bouvard V, Baan R, Straif K, Grosse Y, Secretan B, ElGhissassi F (2009). WHO international agency for research on cancer monograph working group: a review of human carcinogens–part B: biological agents. Lancet Oncol.

[3] Doorbar J, Quint W, Banks L, Bravo I, Stoler M, Broker T (2012). The biology and life-cycle of human papillomaviruses. Vaccine.

[4] Guan P, Howell-Jones R, Li N, Bruni L, de Sanjose S, Franceschi S (2012). Human papillomavirus types in 115,789 HPV-positive women: a meta-analysis from cervical infection to cancer. Int J Canc.

[5] Kirnbauer R, Booy N, Chengt DR, Lowy DR, Schiller JT (1992). Papillomavirus Li major capsid protein self-assembles into virus-like particles that are highly immunogenic. Proc Natl Acad Sci USA.

[6] Hagensee ME, Yaegashi N, Galloway DA (1993). Self-assembly of human papillomavirus type 1 capsids by expression of the L1 protein alone or by coexpression of the L1 and L2 capsid proteins. J Virol.

[7] Harper DM, Franco EL, Wheeler CM, Moscicki AB, Romanowski B, Roteli-Martins CM (2006). Sustained efficacy up to 4.5 years of a bivalent L1 virus-like particle vaccine against human papillomavirus types 16 and 18: follow-up from a randomised control trial. Lancet.

[8] FUTURE II Study Group (2007). Prophylactic efficacy of a quadrivalent human papillomavirus (HPV) vaccine in women with virological evidence of HPV infection. J Infect Dis.

[9] Hung CF, Ma B, Monie A, Tsen SW, Wu TC (2008). Therapeutic human papillomavirus vaccines: current clinical trials and future directions. Expert Opin Biol Ther.

[10] Smotkin D, Wettstein F (1986). Transcription of human papillomavirus type 16 early genes in cervical cancer and cancer-derived cell line and identification of the E7 protein. Proc Natl Acad Sci USA.

[11] Androphy EJ, Hubbert NL, Schiller JT, Lowy DR (1987). Identification of the HPV-16 E6 protein from transformed mouse cells and human cervical carcinoma cells. EMBO J.

[12] Hawley-Nelson P, Vousden KH, Hubbert NL, Lowy DR, Schiller JT (1989). HPV16 E6 and E7 proteins cooperate to immortalize human foreskin keratinocytes. EMBO J.

[13] Goodwin EC, DiMaio D (2000). Repression of human papillomavirus oncogenes in HeLa cervical carcinoma cells causes the orderly reactivation of dormant tumor suppressor pathways. Proc Natl Acad Sci USA.

[14] Yoshinouchi M, Yamada T, Kizaki M, Fen J, Koseki T, Ikeda Y (2003). In vitro and in vivo growth suppression of human papillomavirus 16-positive cervical cancer cells by E6 siRNA. Mol Ther.

[15] Song S, Liem A, Miller JA, Lambert PF (2000). Human papillomavirus type 16 E6 and E7 contribute differently to carcinogenesis. Virol.

[16] Eiben GL, Da Silva DM, Fausch SC, Le Poole IC, Nishimura MI, Kast WM (2003). Cervical cancer vaccines: recent advances in HPV research. Viral Immunol.

[17] Meneguzzi G, Cerni C, Kieny MP, Lathe R (1991). Immunization against human papillomavirus type 16 tumor cells with recombinant vaccinia viruses expressing E6 and E7. Virol.

[18] Campo MS, Grindlay GJ, O’Neil BW, Chandrachud LM, McGarvie GM, Jarrett WF (1993). Prophylactic and therapeutic vaccination against a mucosal papillomavirus. J Gen Virol.

[19] Chen L, Mizuno MT, Singhal MC, Hu SL, Galloway DA, Hellström I (1992). Induction of cytotoxic T lymphocytes specific for a syngeneic tumor expressing the E6 oncoprotein of human papillomavirus type 16. J Immunol.

[20] van der Burg SH, Kwappenberg KM, O’Neill T, Brandt RM, Melief CJ, Hickling JK (2001). Pre-clinical safety and efficacy of TA-CIN, a recombinant HPV16 L2E6E7 fusion protein vaccine, in homologous and heterologous prime-boost regimens. Vaccine.

[21] Chen CH, Wang TL, Ji H, Hung CF, Pardoll DM, Cheng WF (2001). Recombinant DNA vaccines protect against tumors that are resistant to recombinant vaccinia vaccines containing the same gene. Gene Ther.

[22] Porgador A, Irvine KR, Iwasaki A, Barber BH, Restifo NP, Germain RN (1998). Predominant role for directly transfected dendritic cells in antigen presentation to CD8+ T cells after gene gun immunization. J Exp Med.

[23] Hsieh CJ, Kim TW, Hung CF, Juang J, Moniz M, Boyd DA (2004). Enhancement of vaccinia vaccine potency by linkage of tumor antigen gene to gene encoding calreticulin. Vaccine.

[24] Lamikanra A, Pan ZK, Isaacs SN, Wu TC, Paterson Y (2001). Regression of established human papillomavirus type 16 (HPV-16) immortalized tumors in vivo by vaccinia viruses expressing different forms of HPV-16 E7 correlates with enhanced CD8(+) T-cell responses that home to the tumor site. J Virol.

[25] Borysiewicz LK, Fiander A, Nimako M, Man S, Wilkinson GW, Westmoreland D (1996). A recombinant vaccinia virus encoding human papillomavirus types 16 and 18, E6 and E7 proteins as immunotherapy for cervical cancer. Lancet.

[26] Adams M, Borysiewicz L, Fiander A, Man S, Jasani B, Navabi H (2001). Clinical studies of human papilloma vaccines in pre-invasive and invasive cancer. Vaccine.

[27] Baldwin PJ, van der Burg SH, Boswell CM, Offringa R, Hickling JK, Dobson J (2003). Vaccinia-expressed human papillomavirus 16 and 18 e6 and e7 as a therapeutic vaccination for vulval and vaginal intraepithelial neoplasia. Clin Cancer Res.

[28] Smyth LJ, Van Poelgeest MI, Davidson EJ, Kwappenberg KM, Burt D, Sehr P (2004). Immunological responses in women with human papillomavirus type 16 (HPV-16)-associated anogenital intraepithelial neoplasia induced by heterologous prime-boost HPV-16 oncogene vaccination. Clin Cancer Res.

[29] Acres B, Bonnefoy JY (2008). Clinical development of MVA-based therapeutic cancer vaccines. Expert Rev Vaccines.

[30] Brun JL, Dalstein V, Leveque J, Mathevet P, Raulic P, Baldauf JJ, Scholl S, Huynh B, Riethmuller D, Clavel C, Birembaut P, Calenda V, Baudin M, Bory JP (2011). Regression of high-grade cervical intraepithelial neoplasia with TG4001 targeted immunotherapy. Am J Obstet Gynecol.

[31] Chen CH, Wang TL, Hung CF, Pardoll DM, Wu TC (2000). Boosting with recombinant vaccinia increases HPV-16 E7-specific T cell precursor frequencies of HPV-16 E7-expressing DNA vaccines. Vaccine.

[32] Mackova J, Stasikova J, Kutinova L, Masin J, Hainz P, Simsova M (2006). Prime/boost immunotherapy of HPV16-induced tumors with E7 protein delivered by Bordetella adenylate cyclase and modified vaccinia virus Ankara. Cancer Immunol Immunother.

[33] Wlazlo AP, Deng H, Giles-Davis W, Ertl HC (2004). DNA vaccines against the human papillomavirus type 16 E6 or E7 oncoproteins. Cancer gene Ther.

[34] Fiander AN, Tristram A, Davidson EJ, Tomlinson AE, Man S, Baldwin PJ (2006). Prime-boost vaccination strategy in women with high-grade, noncervical anogenital intraepithelial neoplasia: clinical results from a multicenter phase II trial. Int J Gynecol Cancer.

[35] Blanchard TJ, Alcami A, Andrea P, Smith GL (1998). Modified vaccinia virus Ankara undergoes limited replication in human cells and lacks several immunomodulatory proteins: implications for use as a human vaccine. J Gen Virol.

[36] Drexler I, Heller K, Wahren B, Erfle V, Sutter G (1998). Highly attenuated modified vaccinia virus Ankara replicates in baby hamster kidney cells, a potential host for virus propagation, but not in various human transformed and primary cells. J Gen Virol.

[37] Radaelli A, De Giuli Morghen C, Zanotto C, Pacchioni S, Bissa M, Franconi R (2012). A prime/boost strategy by DNA/fowlpox recombinants expressing a mutant E7 protein for the immunotherapy of HPV-associated cancers. Virus Res.

[38] Taylor J, Paoletti E (1988). Fowlpox virus as a vector in non-avian species. Vaccine.

[39] Bissa M, Pacchioni S, Zanotto C, De Giuli Morghen C, Radaelli A (2013). GFP co-expression reduces the A33R gene expression driven by a fowlpox vector in replication permissive and non-permissive cell lines. J Virol Methods.

[40] Pacchioni S, Bissa M, Zanotto C, De Giuli Morghen C, Illiano E, Radaelli A (2013). L1R, A27L, A33R and B5R vaccinia virus genes expressed by fowlpox recombinants as putative novel orthopoxvirus vaccines. J Transl Med.

[41] Nominé Y, Masson M, Charbonnier S, Zanier K, Ristriani T, Deryckere F (2006). Structural and functional analysis of E6 oncoprotein: insights in the molecular pathways of human papillomavirus-mediated pathogenesis. Mol Cell.

[42] Ristriani T, Fournane S, Orfanoudakis G, Masson M (2009). A single-codon mutation converts HPV16 E6 oncoprotein into a potential tumor suppressor, which induces p53-dependent senescence of HPV-positive HeLa cervical cancer cells. Oncogene.

[43] Venuti A, Massa S, Mett V, Vedova LD, Paolini F, Franconi R (2009). An E7-based therapeutic vaccine protects mice against HPV16 associated cancer. Vaccine.

[44] Pacchioni S, Volonté L, Zanotto C, Pozzi E, De Giuli Morghen C, Radaelli A (2010). Canarypox and fowlpox viruses as recombinant vaccine vectors: an ultrastructural comparative analysis. Arch Virol.

[45] Rosel JL, Earl PL, Weir J, Moss B (1986). Conserved TAAATG sequence at the transcriptional and translational initiation sites of vaccinia virus late genes deduced by structural and functional analysis of the hindlll H genome fragment. J Virol.

[46] Pozzi E, Basavecchia V, Zanotto C, Pacchioni S, De Giuli Morghen C, Radaelli A (2009). Construction and characterization of recombinant fowlpox viruses expressing human papilloma virus E6 and E7 oncoproteins. J Virol Methods.

[47] Radaelli A, De Giuli Morghen C (1994). Expression of HIV-1 envelope gene by recombinant avipoxvirus. Vaccine.

[48] Zanotto C, Pozzi E, Pacchioni S, Bissa M, De Giuli Morghen C, Radaelli A (2011). Construction and characterisation of a recombinant fowlpox virus that expresses the human papilloma virus L1 protein. J Transl Med.

[49] Pozzi E, Zanotto C, Pacchioni S, De Giuli Morghen C, Radaelli A (2009). MHC-restricted CTL assay: an improved method based on naïve and SV40-immortalized rabbit epidermal target cells. J Virol Methods.

[50] Radaelli A, Pozzi E, Pacchioni S, Zanotto C, De Giuli Morghen C (2010). Fowlpox virus recombinants expressing HPV-16 E6 and E7 oncogenes for the therapy of cervical carcinoma elicit humoral and cell-mediated responses in rabbits. J Transl Med.

[51] Bissa M, Pacchioni S, Zanotto C, De Giuli Morghen C, Illiano E, Granucci F (2013). Systemically administered DNA and fowlpox recombinants expressing four vaccinia virus genes although immunogenic do notprotect mice against the highly pathogenic IHD-J vaccinia strain. Virus Res.

[52] Radaelli A, Bonduelle O, Beggio P, Mahe B, Pozzi E, Elli V (2007). Prime-boost immunization with DNA, recombinant fowlpox virus and VLP(SHIV) elicit both neutralizing antibodies and IFNgamma-producing T cells against the HIV-envelope protein in mice that control env-bearing tumour cells. Vaccine.

[53] Peng S, Ji H, Trimble C, He L, Tsai YC, Yeatermeyer J (2004). Development of a DNA vaccine targeting human papillomavirus type 16 oncoprotein E6. J Virol.

[54] Jochmus I, Osen W, Altmann A, Buck G, Hofmann B, Schneider A (1997). Specificity of human cytotoxic T lymphocytes induced by a human papillomavirus type 16 E7-derived peptide. J Gen Virol.

[55] Nominé Y, Charbonnier S, Ristriani T, Stier G, Masson M, Cavusoglu N (2003). Domain substructure of HPV E6 oncoprotein: biophysical characterization of the E6 C-terminal DNA-binding domain. Biochemistry.

[56] Sedman SA, Barbosa MS, Vass WC, Hubbert NL, Haas JA, Lowy DR (1991). The full-length E6 protein of human papillomavirus type 16 has transforming and trans-activating activities and cooperates with E7 to immortalize keratinocytes in culture. J Virol.

[57] Mizuuchi M, Hirohashi Y, Torigoe T, Kuroda T, Yasuda K, Shimizu Y (2012). Novel oligomannose liposome-DNA complex DNA vaccination efficiently evokes anti-HPV E6 and E7 CTL responses. Exp Mol Pathol.

[58] Kanodia S, Da Silva DM, Kast WM (2008). Recent advances in strategies for immunotherapy of human papillomavirus-induced lesions. Int J Cancer.

[59] Gissmann L. Modern uterine cytopathology. Meisels A and Morin C, editors. Chicago: ASCP Press. 2007;169–200.

[60] Meyer SI, Fuglsang K, Blaakaer J. Cell-mediated immune response: a clinical review of the therapeutic potential of human papillomavirus vaccination. Acta Obstet Gynecol Scand. 2014. doi:10.1111/aogs.12480. 10.1111/aogs.12480 25146484

[61] Peng S, Song L, Knoff J, Wang JW, Chang YN, Hannaman D (2014). Control of HPV-associated tumors by innovative therapeutic HPV DNA vaccine in the absence of CD4+ T cells. Cell Biosci.

[62] Brandsma JL, Shlyankevich M, Su Y, Zelterman D, Rose J, Buonocore L (2010). Reversal of papilloma growth in rabbits therapeutically vaccinated against E6 with naked DNA and/or vesicular stomatitis virus vectors. Vaccine.

[63] Brandsma JL, Shlyankevich M, Zelterman D, Su Y (2014). Therapeutic vaccination of rabbits with a ubiquitin-fused papillomavirus E1, E2, E6 and E7 DNA vaccine. Vaccine.

[64] Bagarazzi ML, Yan J, Morrow MP, Shen X, Parker RL, Lee JC, Giffear M, Pankhong P, Khan AS, Broderick KE, Knott C, Lin F, Boyer JD, Draghia-Akli R, White CJ, Kim CJ, Weiner DB, Sardesai NY (2012). Immunotherapy against HPV16/18 generates potent TH1 and cytotoxic cellular immune responses. Sci Transl Med.

[65] Massa S, Simeone P, Muller A, Benvenuto E, Venuti A, Franconi R (2008). Antitumor activity of DNA vaccines based on the Human Papillomavirus-16 E7 protein genetically fused to a plant virus coat protein. Hum Gene Ther.

[66] Whitehead M, Ohlschläger P, Almajhdi FN, Alloza L, Marzábal P, Meyers AE (2014). Human papillomavirus (HPV) type 16 E7 protein bodies cause tumour regression in mice. BMC Cancer.

[67] Skinner MA, Laidlaw SM, Eldaghayes I, Kaiser P, Cottingham MG (2005). Fowlpox virus as a recombinant vaccine vector for use in mammals and poultry. Expert Rev Vaccines.

